# Demonstration of the Use of NSGA-II for Optimization of Sparse Acoustic Arrays

**DOI:** 10.3390/s25185882

**Published:** 2025-09-19

**Authors:** Christopher E. Petrin, Trevor C. Wilson, Aaron S. Alexander, Brian R. Elbing

**Affiliations:** 1 Mechanical and Aerospace Engineering, Oklahoma State University, Stillwater, OK 74078, USA; cepetri@okstate.edu (C.E.P.); trevor.wilson@okstate.edu (T.C.W.); aaral@okstate.edu (A.S.A.); 2 Mechanical Engineering Technology, Oklahoma State University, Stillwater, OK 74078, USA

**Keywords:** array response, beamforming, infrasound, NSGA-II

## Abstract

Passive acoustic sensing with arrays has applications in many fields, including atmospheric monitoring of low frequency sounds (i.e., infrasound). Beamforming of array signals to gain spatial information about the signal is common, but the performance is often degraded due to limited resources (e.g., number of sensors, array size). Such sparse arrays create ambiguities due to reduced resolution and spatial aliasing. While previous work has focused on either maximizing array resolution or minimizing spatial aliasing, the current study demonstrates how evolutionary algorithms can be utilized to identify array configurations that optimize for both properties. The non-dominated sorting genetic algorithm II (NSGA-II) was used with the beamwidth and maximum sidelobe level as the fitness functions to iteratively identify a group of optimized synthesized array configurations. This group is termed a Pareto-front and is optimized such that one fitness function cannot be improved without a decrease in the other. These optimized solutions were studied for a single frequency (8 Hz) and a multi-frequency (3 to 20 Hz) signal using either a 36-element or 9-element array with a 60 m aperture. The performance of the synthesized arrays was compared against established array configurations (baseline) with most of the Pareto-front solutions outperforming these baseline configurations. The largest improvements to array performance using the synthesized configurations were with fewer array elements and the multi-frequency signal.

## 1. Introduction

Passive acoustic sensing is widely used in many applications, including seismology [1,2,3,4,5], severe weather monitoring [6,7,8,9,10], explosions [11,12,13,14], uncrewed systems detection [15], and atmospheric entries [16,17,18,19,20,21]. While single sensors are common (e.g., [22,23]), multi-element (i.e., sensor) arrays are preferrable since they can provide spatial/directional information about the received signals using beamforming (e.g., [24,25,26]). However, the beamforming performance is highly dependent on the arrangement of the array elements [27]. Ideally, the array would have an infinite number of omnidirectional elements [28], but for practical reasons (e.g., cost, available space, hardware performance, and processing requirements), acoustic arrays have limited performance [29]. Thus, arrays always sample discretely in time and space and, as such, yield spatial information that can be ambiguous or impossible to differentiate [27]. This ambiguity is amplified when there is a low quantity of elements (i.e., sparse array). In addition to the number of elements, the beamforming performance is primarily dependent on the geometric configuration of the array elements and the frequency band of the received signal. These properties often lead to competing interests in array design (e.g., minimizing the array footprint will also restrict the frequency band that can be detected). System identification methods and compressed sensing can both be used in the processing and post-processing of recorded signals to identify the number of sources in a signal [30] and estimate directional information from a sparsely sampled signal [31], respectively. However, some ambiguities in beamforming can be mitigated by optimizing the geometric array configuration. The focus of the current work is to demonstrate how evolutionary algorithms can be utilized to optimize the geometric positioning of the elements in a sparse array.

Monitoring atmospheric infrasound (i.e., sound at frequencies below the nominal threshold of human hearing, 20 Hz) is an application that commonly utilizes sparse arrays with element positions constrained due to the relatively large apertures (i.e., largest spacing between elements). For example, the Infrasound Monitoring System (IMS) stations monitor infrasound between 0.02 Hz and 4 Hz [32,33] with a recommended aperture of 3 km [34] though they often have less than 12 elements. Many other infrasound observations have used less elements (e.g., [7,8,9,10]). Even the Source Physics Experiment, [35,36] that had a relatively large array (up to 32 elements), includes array observations with as few as 4 elements. Consequently, infrasound monitoring is taken as the representative example problem for the current study, though the concepts are applicable to any array design.

The use of evolutionary algorithms requires a set of metrics to quantify the quality of a given array design. The array response pattern (i.e., array beampattern [37,38], array transfer function [38,39], or spatial window function [1]) can be derived from frequency-wavenumber spectrum analysis [40]. For a given angular frequency (ω=2πf), the array response (R) is defined within a two-dimensional domain termed the slowness grid using the slowness vector ($\vec{p}=\left(k_{x},k_{y}\right)/\omega$), where $\vec{k}=\frac{\omega }{c}$ is the wavenumber at a given position ${\vec{r}}_{n}=\left(x_{n},y_{n}\right)$ and f is the frequency. The magnitude of the slowness vector is equal to the inverse of the apparent sound speed ($c_{app}$), and the back azimuth (ϕ) sets the vector components $\left(p_{x}=\cos\left(\phi \right)/c_{app},\;{\;p}_{y}=\sin\left(\phi \right)/c_{app}\right)$. Here, the apparent sound speed is the speed of the signal moving across the array, which is a function of the inclination angle (θ) between the plane of the array and the incoming signal, such that a horizontally propagating wave has an inclination angle of 0° for a level ground array. For acoustic waves, the apparent sound speed is the effective sound speed (sum of the thermodynamic sound speed and the wind speed projected in the direction of interest [41]) divided by the cosine of the inclination angle, $c_{app}\;=\;c_{eff}/\cos\left(\theta \right)$. The current work follows the array response analysis described in Evers [28], which assumes plane wave propagation in a locally homogeneous medium with the array response of

(1) $$R\left(\omega ,\vec{p},\vec{r}\right)={\left|\frac{1}{N}\sum_{n=1}^{N}\exp\left(-i\omega \left[\left(\vec{p}-{\vec{p}}_{0}\right)\cdot {\vec{r}}_{n}\right]\right)\right|}^{2}.$$

here, *N* is the number of sensors, i is the imaginary unit, and ${\vec{p}}_{0}$ is a reference wave input that is used when beamforming a known slowness (neglected when deriving a general response). Following typical convention, the reported *R* values in the current work are normalized using the value at $\vec{p}$ = (0, 0).

**Figure 1:**
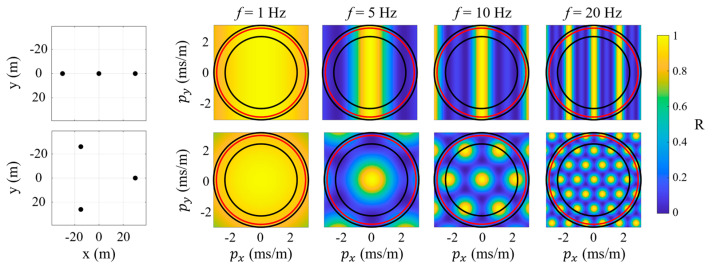
To illustrate the frequency sensitivity of the array response to configuration and aperture, array responses are shown for 3-element arrays with an aperture of 60 m in a (**top row**) linear and (**bottom row**) equilateral triangle configuration for signals at 1, 5, 10, and 20 Hz. The array configurations are shown on the left with the coordinate origin at the array center. The concentric circles correspond to apparent sound speeds of (inner) 420 m/s, (middle) 343 m/s, and (outer) 320 m/s.

To illustrate the array response, the array responses of two different 3-element arrays (linear and equilateral triangle) are plotted in Figure 1 for different signal frequencies (1, 5, 10, and 20 Hz). The ideal array response pattern would be a peak at the origin, $\vec{p}=\left(p_{x},p_{y}\right)=\left(0,0\right)$, approximating a delta function and no response at any other point [1]. Bright spots (i.e., larger values of *R*) apart from the origin thus indicate apparent sound speeds $\left(\left|\vec{p}\right|=1/c_{app}\right)$ where the array cannot resolve spatial information. For example, the linear array is asymmetric and consequently its response is directionally dependent with an inability to differentiate between a wave propagating orthogonally to the array (i.e., bright vertical line along $p_{x}=0$). The remainder of the current work will focus on omnidirectional array configurations such as the equilateral triangle configuration. Every array response has a bright spot (termed main lobe) at its origin (i.e., infinite apparent speed), and for omnidirectional arrays the main lobe diameter varies with the configuration and signal frequency. The main lobe diameter is termed the beamwidth (*BW*), and in the current work the *BW* is defined as the main lobe diameter when the prominence decreases by half of its peak (i.e., half-prominence bandwidth). Additional lobes are observed in the array responses of the higher frequency signals, which are termed sidelobes. The sidelobes are an artifact of discrete spatial sampling and are related to the number of repeated inter-element distances [37,42,43]. The maximum sidelobe level (*MSL*) is the maximum response (R) of any sidelobes. Hence, an ideal array response would have an infinitesimal *BW* and zero *MSL*, so these two parameters will serve as the metrics to quantify the quality of a given array design in the current work.

For arrays with a very low number of elements (<5) there are few configurations that are omnidirectional, and the optimal solutions are known (e.g., an equilateral triangle is the only omnidirectional configuration for a 3-element array). However, it is unclear how best to utilize additional elements for sparse arrays with more than ~5 elements. Thus, the current work aims to demonstrate the use of evolutionary algorithms to optimize (i.e., minimize *BW* and *MSL*) element positioning for a fixed number of elements in a sparse array with a given aperture (*D*) while maintaining omnidirectionality. The remainder of the paper includes a description of the methodology in Section 2, results for reference array configurations and newly generated arrays are presented in Section 3, discussion of the use of such optimization algorithms in Sec. IV, and conclusions drawn in Section 4.

## 2. Methodology

### 2.1. Array Design

#### 2.1.1. Design Criteria

For a given source (i.e., fixed frequency band of interest), the array response is controlled by the number of sensors (*N*), the aperture (*D*), and the geometric configuration of the elements (${\vec{r}}_{i}$). It is always desirable to maximize *N*, but its value is typically constrained by resource availability. Hence, sensor number is not considered a design choice, but different values of *N* will be considered in the current analysis. The main lobe beamwidth (*BW*) is inversely proportional to the array aperture for a given frequency and decreases with increasing frequency. For a fixed number of sensors and aperture, the array configuration (${\vec{r}}_{i}$) is the only means to mitigate sidelobes, which typically is achieved by reducing the number of repeated inter-element distances [37]. Following the work of Evers [28], the current work uses the following performance criteria for omnidirectional detection of a signal at a given frequency:

1. Main lobe should be very thin to maximum resolution (i.e., minimize *BW*).
2. Sidelobe amplitudes should be small to avoid spatial aliasing (i.e., minimize *MSL*).
3. Main lobe should be circular to ensure equal resolution in all directions.
4. Sidelobes should be kept out of the slowness domain of interest to avoid spatial aliasing.

The implementation of the final criterion is dependent on the expected operational conditions (i.e., wind conditions) and orientation of the received signal, particularly the signal inclination angle. For example, a horizontally propagating wave with no wind would have an apparent sound speed of ~343 m/s, which corresponds to a slowness magnitude of $2.9\times 10^{-3}\;s/m$. For reference, this is marked in Figure 1 with a red circle on each array response. For the current work, the domain of interest spanned apparent sound speeds from 320 m/s to infinity. Infinite apparent sound speed (i.e., slowness magnitude of 0 s/m) corresponds to a vertically downward propagating wave. The minimum apparent sound speed was selected to match Wilson et al. [10], which 320 m/s (e.g., θ=0° with a 23 m/s head wind) corresponds to a slowness magnitude of $3.1\times 10^{-3}\;s/m$. For reference, additional lines in Figure 1 mark the 320 m/s apparent sound speed and 420 m/s (e.g., θ=30° with a 20 m/s tail wind), which was the upper limit in Wilson et al. [10] that will be used subsequently. The grid was formed of 100 uniformly distributed points from the minimum to maximum slowness. Thus, evaluation of sidelobes in the current study will be based on values within the slowness grid spanning apparent sound speeds of 320 m/s and infinity. The array encoding (see below) forced main lobe symmetry, which allowed the main lobe beamwidth to be calculated as the maximum half-prominence width within a 45° slice of the slowness grid. However, this method would not be suited to highly skewed main lobes. The half-prominence widths were computed by linear interpolation along the raw slowness contour.

#### 2.1.2. Array Encoding

The coordinates of each array element $\left({\vec{r}}_{i}=\left(x_{i},y_{i}\right)\right)$ are the decision variables since they determine the array configuration and performance at a given frequency. The third design criterion (circular main lobe) restricts the quantity of viable solutions and can be enforced by requiring symmetry of the array element locations over more than two planes of symmetry. This is achieved in the current work following the method of Bjelić et al. [37] that divides the array into *S* slices of equal angle, selecting the element configuration within one slice/segment, and then copying that arrangement in the remaining segments. Using polar coordinates, the element placement is constrained between 0 and 2π/S radians with a radius ≤D/2. A minimum inter-element distance of 1 m was prescribed to account for limitations imposed by sensors of finite size. In addition, a radius of zero was not allowed since it would replicate *S* elements at the center; rather, array designs were divided between configurations with and without center elements. In the current work, the aperture of an array is the maximum inter-element distance, and the maximum aperture allowed was 60 m. This value was arbitrary (though it needs to be set based on the expected signal frequency) and selected because it was the nominal size of a widely used array [8,10,20]. As an aside, this method is readily adaptable for array deployments on parcels of land with obstructions (e.g., fences, buildings, etc.) by mapping the obstructions into the 2π/S radian segment and disallowing placement of elements within that space.

The number of decision variables encoded changes based on the selection of *N*, *S*, and if a center element is used, but the genetic analogy requires that each “species” have the same gene length (i.e., number of decision variables). Consequently, the algorithm cannot be run for all species (i.e., all combinations of *N*, *S*, and with/without center element) simultaneously because the genes must be encoded in their own solution pool. Thus, different species (i.e., combination of *N*, *S*, and with/without center element) will be individually analyzed and then the performance between species will be compared. Not all combinations are possible due to the requirement that an integer number of elements must be within each segment, where each segment has $\left(N-1\right)/S$ and N/S elements for configurations with and without center elements, respectively. This method also reduces the computational cost by reducing the decision variables (i.e., element positions) from 2N to 2N/S (or $2\left(N-1\right)/S$). For the current demonstration, five different species are considered, and they are listed in Table 1. The number of elements examined (9 and 36) were selected such that there were an intermediate number of elements (i.e., above minimum while still sparse) that allow configurations with and without center elements.

#### 2.1.3. Reference Array Configurations

For comparison, nine “baseline” array configurations were identified and used as a reference to compare the performance of the derived array configurations. Each array had a 60 m aperture and mimicked key characteristics of arrays identified in the literature. Table 2 lists the nine baseline array configurations selected with 9 or 36 elements. The configurations are a uniform rectangular grid, a uniformly spaced circle (with and without a center element), a constant density concentric circle (equal number of elements in each circle), an Arcondoulis spiral [44], a log-based multispiral, an Underbrink multispiral [45,46], and two variable-density concentric polygon (VDCP) arrays. The selection of baseline arrays was made to represent common solutions to array geometry configuration. The uniform grid, uniformly spaced circle and constant density concentric circle arrays were selected because they are commonly used as theoretical examples [47]. The spiral and multispiral arrays were selected as popular non-uniform but formulaic arrays [48]. Finally, the VDCP arrays were inspired by the geometries of various IMS stations [34]. Many of these array configurations have variable input parameters, but no attempt was made in the current study to optimize these input parameters for the particular application. Rather, the parameters were selected following a previous array design comparison study [48] and are also listed in Table 2. Hence, the results are not reflections of the quality of these designs but rather provide a reference range if picking established designs without performing some optimization similar to what is described in this study.

### 2.2. Optimization Algorithm

#### 2.2.1. Overview of NSGA-II

The fundamental hypothesis of this work is that array geometry design should be treated as a multi-objective optimization problem (MOP) between improving resolution (i.e., minimizing BW) and reducing spatial aliasing (i.e., minimizing MSL). The use of genetic algorithms to synthesize antenna array designs has been conducted [49,50], but these studies were not focused on identifying optimal sparse array configurations but rather identifying which sensors within a large array (hundreds of elements) should be utilized in their processing. Such an approach is not feasible for most low-frequency acoustic sensing applications where cost and space constraints limit the practical number of sensors and require that decisions be made a priori. For the current work, the non-dominated sorting genetic algorithm II (NSGA-II) [51] was chosen because it implements fast non-dominated sorting to explore Pareto-efficient solutions to MOPs, particularly those with two fitness functions (e.g., minimizing *BW* and *MSL*). It is one of the most popular and widely implemented algorithms for solving MOPs [52] and offers a relatively straightforward approach to optimizing the array element locations as encoded.

**Figure 2:**
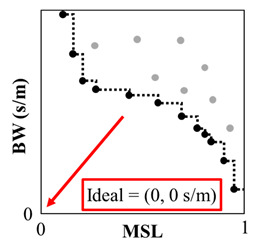
An illustration of the expected results of the NSGA-II optimization process for array design. The previous generation of array configurations (gray) is used as the basis for generating a new set of array designs (black). A Pareto-front is formed (black dashed line) and, over a number of generations, the new fronts should trend toward the ideal of (MSL, BW) = (0, 0 s/m). The read arrow points to the ideal design condition.

NSGA-II works iteratively by encoding solutions into a set of decision variables that are evaluated using the two fitness functions and sorted into groups of Pareto-fronts. After grouping the encoded solutions, the next iteration is generated by combining pairs of encoded solutions and randomly changing parts of the resulting combined solutions. By analogy, the encoded solutions are called “genes,” each iteration of encoded solutions is called a “generation,” and the combination and random change in parts of the new genes are called “crossover” and “mutation,” respectively. For the current study, the genes are groups of array designs that NSGA-II creates using the fitness functions (*BW* and *MSL*) to iteratively synthesize new array designs. When the solution performance with respect to one fitness function cannot be improved without a decrease in performance of the other fitness function, this is termed a Pareto-efficient condition. Solutions grouped such that each solution is Pareto-efficient compared to the others are called a Pareto-front. This process is sketched in Figure 2 with previous generations shown in gray while the current iteration is in black, which is moving towards the ideal configuration (i.e., the origin).

#### 2.2.2. Implementation of NSGA-II

For the current study, each species (set of array design parameters) in Table 1 uses NSGA-II to synthesize arrays in non-dominated fronts with 1000 genes in each generation, for a maximum of 1000 generations. Each gene real-codes the polar coordinates of a single array segment. The azimuthal coordinates are limited between 0 and 2π/S radians, and the radial coordinates are constrained from 1 m to 30 m (i.e., 60 m aperture) with a minimum of 1 m separation between elements. When a new solution is generated that violates the minimum spacing, an entirely new gene was generated at random that fulfills all constraints but is not related to the previously selected parents. The next generation was generated using 20% of the gene pool size. The gene pairs were selected by binary tournament [53], where two random genes were compared for each available parent pool slots. To encourage solution diversity, crowded solutions (i.e., solutions that were near each other on a Pareto-front) were penalized based upon the average separation between adjacent solutions. The candidate was selected if it was in the more dominant Pareto-efficient front. If on the same front, the gene furthest from other solutions was selected, but if there was equal separation the first was selected. New genes were created by simulated binary crossover [54] with a crossover distribution index of 20. Each gene created also had a chance equal to the inverse number of decision variables to undergo polynomial mutation following Deb and Agrawal [55], with a mutation index of 20. A thousand child genes were created, evaluated, and non-dominated sorted together with the previous generation. From the sorted intermediate pool containing the parent generation and the child generation, the top 1000 genes were selected as the new Pareto front. For a single frequency, minimizing the *BW* and *MSL* were the two fitness functions. When considering a frequency band, the current study a set of discrete frequencies (3, 10, 13, 20 Hz), calculated their individual *BW* and *MSL* values, and then the mean *BW* and *MSL* values were used as fitness functions. The minimum frequency (3 Hz) was selected due to the previously noted side lobes in the array of Wilson et al. [10], and the maximum frequency is the nominal upper-bound of the infrasound regime. The two intermediate frequencies were selected such that they had nominally equal logarithmic spacing. The use of the mean value for the fitness function is only an example of a metric that could be used. It is important to note that this potentially obscures weaknesses at specific frequencies. Such concerns could be mitigated by using a finer frequency resolution as well as implementing a custom metric that also incorporates the standard deviation.

## 3. Results

### 3.1. Baseline Array Performance

**Figure 3:**
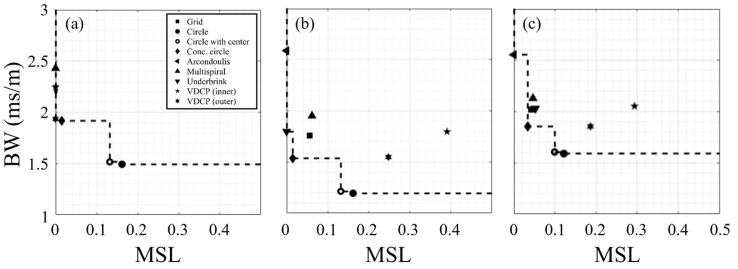
Comparison of performance and Pareto-fronts for 36-element baseline arrays with (**a**) 8 Hz, (**b**) 10 Hz, and (**c**) multi-frequency (3, 10, 13, and 20 Hz) signals. The BW and MSL for the multi-frequency results are mean values. Dashed lines are the Pareto-front formed for each set of arrays.

The performance of the 36-element baseline array configurations (see Table 2) for an 8 Hz, 10 Hz, and multi-frequency (3, 10, 13, and 20 Hz) signal are plotted in Figure 3. The Pareto-front formed by this set of baseline array configurations is marked with dashed lines for each signal. For example, the 8 Hz baseline Pareto-front is formed with VDCP (outer), concentric circle, and circle arrays (with and without a center element). When the signal frequency increased to 10 Hz, the Pareto-front changes to the Underbrink mutispiral, concentric circle, and circle arrays (with and without a center element). As expected (see Figure 1), the increase in signal frequency results in a decrease in *BW* with an increase in potential for sidelobes forming within the domain of interest. However, these differences also highlight the potentially high sensitivity of the configuration to the signal of interest. For example, the VDCP (outer) *MSL* increased from 0 to ~0.25 with the signal moving from 8 to 10 Hz, which moved it off the Pareto-front. It is therefore recommended to optimize for the expected frequency range and not merely a center frequency. The multi-frequency results (Figure 3c) illustrate how this can be achieved using the mean *BW* and mean *MSL* determined from the results of individual frequencies to identify the Pareto-front, which was formed from the Arcondoulis spiral, concentric circle, and uniform circle (with and without a center element).

**Figure 4:**
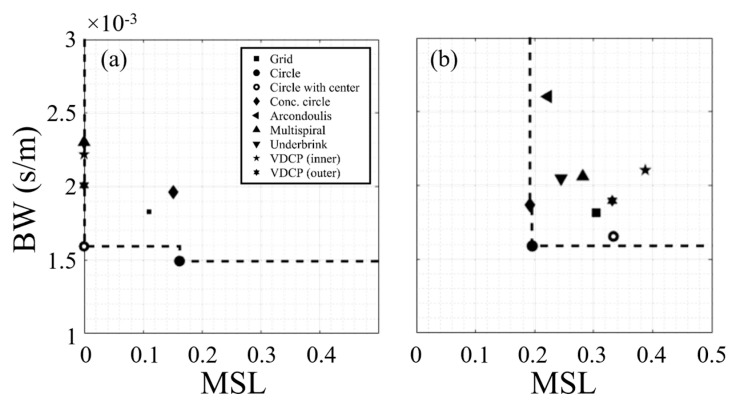
Comparison of performance and Pareto-fronts for the 9-element baseline arrays with (**a**) 8 Hz and (**b**) multi-frequency (3, 10, 13, and 20 Hz) signals. The BW and MSL for the multi-frequency results are mean values. The dashed lines are the Pareto-front formed for each set of arrays.

The performance of the 9-element baseline array configurations (see Table 2) is plotted in Figure 4. The 10 Hz condition was previously included to highlight the potential sensitivity to the signal frequency, but the subsequent analysis will focus on the 8 Hz and multi-frequency signals. For the 8 Hz signal, the Pareto-front is formed from the uniform circle (with and without the center element) configurations. For the multi-frequency signal, the Pareto-front is formed from the concentric circle and uniform circle (without center element) configurations. The degraded *MSL* performance with the multi-frequency signal highlights that the array performance becomes more variable over wider frequency ranges as the number of elements decreases. This reinforces the importance of considering multiple frequencies when optimizing an array configuration, especially as the number of elements decreases. The performance of these baseline configurations will serve as references when comparing the evolutionary configurations.

### 3.2. Optimization of 36-Element Array

**Figure 5:**
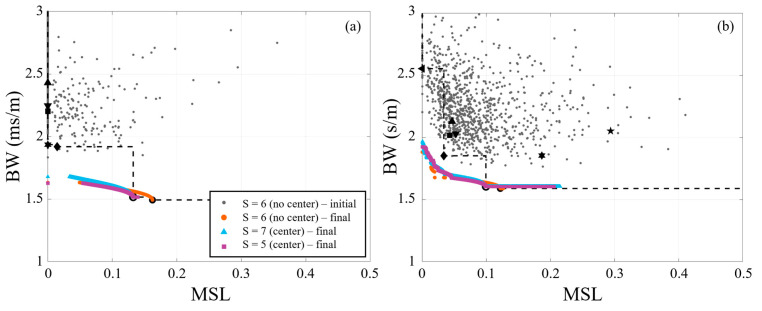
Performance of synthesized 36-element arrays using NSGA-II for (**a**) 8 Hz and (**b**) multi-frequency (3, 10, 13, and 20 Hz) signals. For reference, black markers denote performance of baseline arrays (see Table 2 for marker legend). The black dashed lines mark the baseline Pareto-fronts.

The performance of the 36-element synthesized arrays for the 8 Hz and multi-frequency signals are plotted in Figure 5. Included in the figure is the first generation for one of the species (centerless, S=6), which only one species’ first generation is shown for clarity since all possibilities would obscure individual data points. Some initial synthesized array performances were outside of the figure bounds, but the limits were set to match that used for the reference arrays. The final generation (i.e., 1000th generation) for each 36-element species is also shown. While most of the early generations failed to outperform the baseline Pareto-front, the Pareto-front for the final generation of each synthesized species outperformed the baseline arrays except for the circle arrays (with and without a center). The circle array without a center element is on the Pareto-front from centerless species, and the circle array with a center element is on the Pareto-fronts of the species with a center element. Comparison of the three species shows that their final generations each produced similar performance. The Pareto-fronts for the single and multi-frequency are very similar for *MSL* < ~0.1, but the multi-frequency Pareto-front does not achieve as low of a *BW* (1.5 vs. 1.7) at higher values of *MSL*. Also, the multi-frequency signal shows that the circular reference arrays (center or centerless) mark an inflection point in the front, where for larger *MSL* values there is a negligible decrease in *BW*.

**Figure 6:**
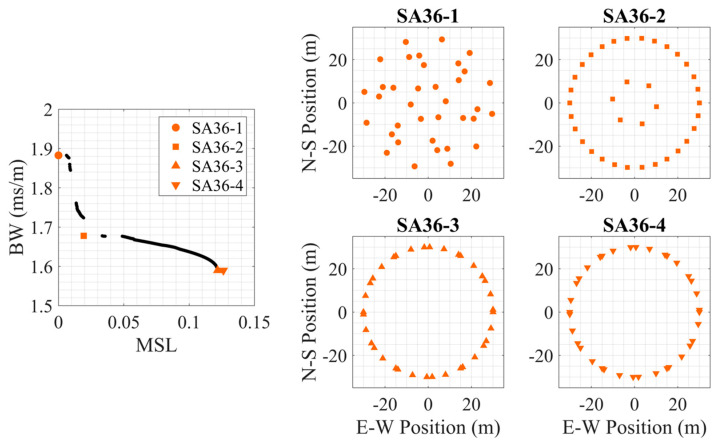
Four 36-element centerless synthesized array (SA36) examples for the multi-frequency signal. (**left**) The Pareto-front for this array species (*N* = 36, *S* = 6, centerless) with the selected array geometries marked, and the synthesized array designs shown on the right. Note that the bounds of the left plot are smaller than those of the other figures.

To better understand the synthesized Pareto-fronts and their corresponding configurations, four array designs on the Pareto-front from a centerless species (*N* = 36, *S* = 6) and a species with a center (*N* = 36, *S* = 7) are further investigated. Three of the synthesized arrays were inflection points on the Pareto-front, and the fourth was the array with the maximum mean *MSL* on the Pareto-front. Figure 6 shows the Pareto-front and the four selected synthesized array (SA) designs, labeled in increasing *MSL* SA36-1, SA36-2, SA36-3, and SA36-4. SA36-3 and SA36-4 are nearly identical with a similar configuration to the centerless circular array design, but the difference is that the element spacing is non-uniform for the synthesized array. The other two arrays also closely resemble baseline designs with SA36-1 similar to multispiral and SA36-2 being similar to VDCP (outer).

**Figure 7:**
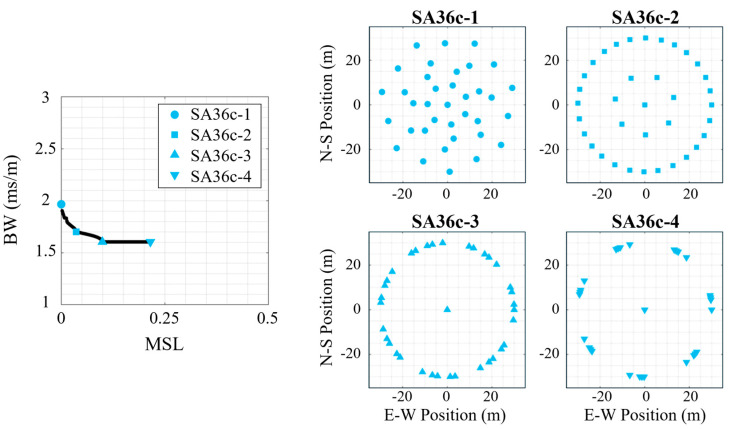
Four 36-element synthesized array (SA36) examples with a center element for the multi-frequency signal. (**left**) The Pareto-front for this array species (*N* = 36, *S* = 7, center) with the selected array geometries marked, and the synthesized array designs shown on the right.

Similarly, the examples on the Pareto-front from the center element species (*N* = 36, *S* = 7) are shown in Figure 7, which the selected configurations having a similar naming convention (SA36c-1 through SA36c-4 with the ‘c’ indicating it has a center element). Once again, the minimum *MSL* design (SA36c-1) had a design similar to the multispiral baseline configuration, and the next lowest *MSL* design (SA36c-2) had a similar design to a VDCP (outer) baseline configuration though with non-uniform inter-element spacing (and a center element). The other two synthesized arrays (SA36c-3 and SA36c-4) once again have distributions that resemble the circular (with center) baseline array configuration with non-uniform element spacing. However, unlike the centerless synthesized results (SA36-3/4) these two examples (SA36c-3/4) have significantly different mean *MSL* values. Inspection of SA36c-4 shows that the elements are grouped so closely together that it appears to be forming a 7-sided polygon rather than a 35-sided one (i.e., a uniformly distributed circular array of *N* elements can be thought of as an N sided polygon). This provides insight into the cause for the flat region with increasing *MSL* without any substantial decrease in *BW*. These configurations represent variants of non-uniform spaced circular arrays. With increasing *N*, a uniform circle array approximates a continuous ring aperture [47]. Creating non-uniform spacing implicitly requires a tighter clustering of elements, which effectively results in a further discretization (that is, worsens the approximation) of the continuous ring aperture. Thus, for a given species of discrete array encoded in radial planes of symmetry, the uniform circle functions as a practical limit to the minimum attainable *BW* as the best approximation possible of the continuous ring aperture.

### 3.3. Optimization of 9-Element Array

**Figure 8:**
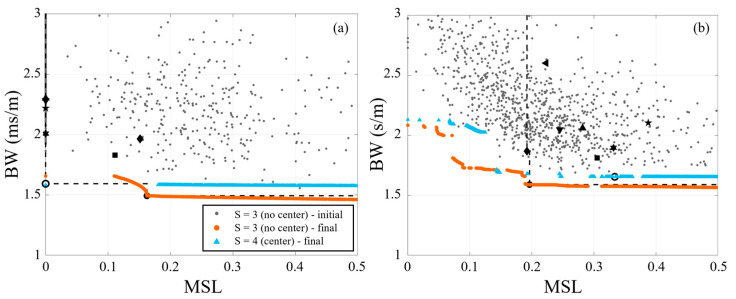
Performance of synthesized 9-element arrays using NSGA-II for (**a**) 8 Hz and (**b**) multi-frequency (3, 10, 13, and 20 Hz) signals. For reference, black markers denote performance of baseline arrays (see Table 2 for marker legend). The black dashed lines mark the baseline Pareto-fronts.

While NSGA-II had some marginal improvements relative to the circle arrays for the relatively large element array, the expectation is that its impact will be greater with more sparse arrays. The performance of the 9-element synthesized arrays for the 8 Hz and multi-frequency signals are shown in Figure 8. Once again, the plots include the first generation for one of the species (centerless, *S* = 3) for clarity and the final generation (i.e., 1000th generation) for both 9-element species. For the single frequency (8 Hz) signal, NSGA-II does not show much improvement relative to the baseline Pareto-front with the circle array marking the inflection point of the *S* = 3 Pareto-front. This reinforces the observation that circular arrays represent the minimum attainable *BW*. The value of the use of NSGA-II is best highlighted with the performance of the sparse (9-element) array with a multi-frequency signal (i.e., the most common scenario for infrasound sensing), shown in Figure 8b. While the circle arrays (center or centerless) still mark the inflection point in the synthesized Pareto-fronts, some of the first generation synthesized arrays achieved lower *MSL* compared to the baseline Pareto-front. The final synthesized Pareto-fronts create unique solutions that will require tradeoff decisions between acceptable levels of *BW* and *MSL*, which will be discussed in more detail subsequently.

**Figure 9:**
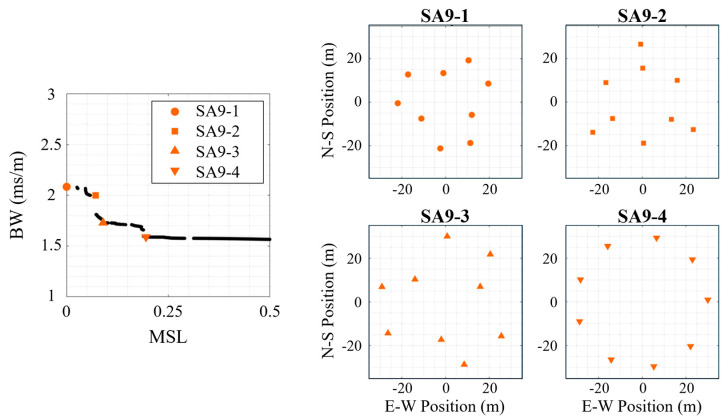
Four 9-element centerless synthesized array (SA9) configurations for the multi-frequency signal. (**left**) The Pareto-front for this array species (*N* = 9, *S* = 3, centerless) with the selected array geometries marked, and the synthesized array designs shown on the right.

As with the 36-element synthesized arrays, a selection of the final generation of the synthesized arrays on the multi-frequency Pareto-front was selected to better understand the Pareto-front configurations. The four selected synthesized arrays for the centerless species (*S* = 3) are shown in Figure 9. The horizontal flat region of the Pareto-front (with *MSL* values greater than that of the circle array) was ignored, and the four arrays were selected from inflection points with lower *MSL* values than the flat region. Following the same naming convention, the 9-element synthesized arrays (SA9) are labeled, in order of increasing *MSL*, SA9-1 through SA9-4. The first two configurations (SA9-1, SA9-2) produced the lowest mean *MSL*, but it appears these arrays achieved this performance by decreasing their apertures (only the maximum aperture was controlled) which has the effect of increasing the size of BW (see BW Figure 1 change with signal frequency). The apertures of SA9-1 and SA9-2 were 42.5 m and 46.0 m, respectively. The fourth configuration (SA9-4) produced performance very close to the baseline uniform circle array, which inspection shows it is the circle array with minor adjustments to produce non-uniform spacing. The other synthesized array (SA9-3) balances these effects by slightly decreasing the aperture (59.4 m) causing a slight increase in *BW* but with a 50% decrease in mean *MSL* relative to the baseline arrays.

**Figure 10:**
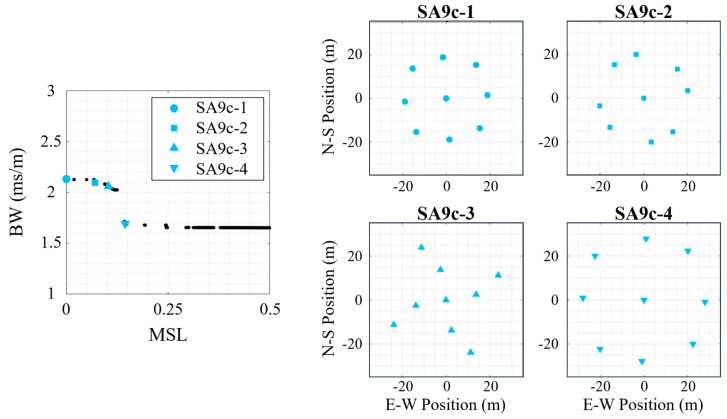
Four 9-element synthesized array (SA9) examples with a center element for the multi-frequency signal. (**left**) The Pareto-front for this array species (*N* = 9, *S* = 4, center) with the selected array geometries marker, and the synthesized array designs shown on the right.

The four selected synthesized arrays for the species with a center element (*N* = 9, *S* = 4, center) are shown in Figure 10 with a similar naming convention (SA9c-1 through SA9c-4). The first three configurations (SA9c-1, SA9c-2, and SA9c-3) all show improved mean *MSL* without improved *BW* due to it being achieved with a reduced aperture (41.1 m, 40.7 m, and 52.8 m, respectively). SA9c-4 has a similar shape to SA9c-1, but SA9c-4 has a larger aperture and consequently produced a significant decrease in *BW* relative to the other synthesized arrays. The outer elements appear to be set along a superelliptic (i.e., squircle) shape in non-uniform increasements, rather than merely a circular shape.

## 4. Discussion

**Figure 11:**
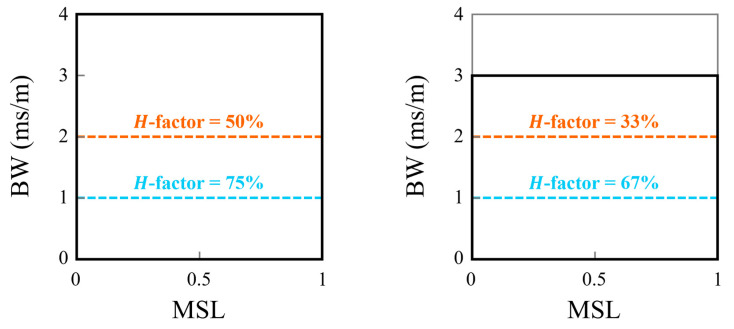
An illustration of the impact of the reference area on the *H*-factor magnitudes. The thick black box marks the reference area, and the dashed (orange and blue) lines mark two different Pareto-fronts. On the left, the reference area fills the entire plot area while on the right it was reduced to a maximum *BW* of 3 ms/m.

The hypervolume parameter (*H*-factor) is a method for quantitative comparison of the Pareto-fronts [56,57]. The H-factor is an estimate of the percentage of a reference area dominated by a Pareto-efficient front. A limitation of this approach is that the reference area is an arbitrary choice and has a direct impact on *H*-factor magnitude. This subjectivity is illustrated in Figure 11 where two plots use different reference areas but identical Pareto-fronts (both with constant *BW*). Here the reduced reference area has a corresponding decrease in the *H*-factor for both Pareto-fronts, but the difference between the curves increases from 25% to 33% with the reduced area. While this subjectivity is well known, the hypervolume indicator is the most common performance metric for MOPs [58]. However, the selection of performance indicators for MOPs remains an open research topic [59]. To mitigate the subjectivity of the current work, a single reference area is utilized. The selected rectangular reference area is bounded between the points (0, 1 × 10^−3^ s/m) and (0.5, 3 × 10^−3^ s/m). This is the same area that is shown in the Pareto-front comparison figures (Figure 3, Figure 4, Figure 5 and Figure 8). This area does not capture all first generations, but it captures the majority of fronts formed for both the baseline and synthesized arrays.

The *H*-factor for each front in the current study was estimated using the method described in Cao [60], which generates a sample of 100,000 random points within the reference area and determines the percentage of these points dominated by the given front. For each Pareto-front, this approach was repeated 300 times, and the resulting ensemble mean and standard deviations are reported in Table 3. Comparisons should only be made within the same signal frequency band, but the *H*-factors can be compared between different species (even with different number of elements). Starting with the single frequency (8 Hz) signal, all 36-element synthesized species outperformed the 36-element baseline front, while none of the 9-element synthesized species outperformed the 9-element baseline front. Further inspection shows that the best 36-element performance (*N* = 36, *S* = 6, centerless) still performed worse than the 9-element baseline front. The excellent performance of the 9-element baseline front hinges on the performance of the circular array with the center, but that array configuration is not even on the baseline Pareto-front when multi-frequency band is considered. This highlights that this approach is likely unnecessary if targeting a signal composed of a single tone when it is undesirable to design arrays targeting a broader frequency band based on a single (e.g., center of frequency band of interest) frequency. For the multi-frequency signal, all synthesized species have higher *H*-factors than the baseline fronts. For the 36-element arrays, the worst performing synthesized front (*N* = 36, *S* = 7, center) had an *H*-factor that was 3% higher than the baseline. These differences were even larger for the 9-element arrays with the worst performing synthesized front (*N* = 9, *S* = 4, center) have an *H*-factor 17% larger than the baseline.

**Figure 12:**
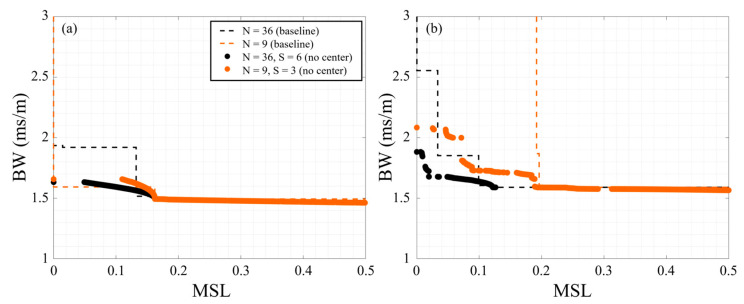
Performance of centerless synthesized arrays using NSGA-II for (**a**) 8 Hz and (**b**) multi-frequency (3, 10, 13, 20 Hz) signals compared with the baseline Pareto-fronts.

Table 3 also shows that for a given signal and number of elements (*N*) the centerless synthesized arrays consistently outperformed the synthesized arrays with a center element. In the extreme case, the centerless 9-element synthesize array (*N* = 9, *S* = 3, centerless) had an *H*-factor that was 5.3 percentage points larger than the 9-element synthesized array with no center element (*N* = 9, *S* = 4, center). It is important to note here that this does not imply that adding a center element degrades the performance of an array, rather it means that if you have an extra sensor placing it at the center is unlikely to be the most effective use of that sensor. For comparison, Figure 12 plots the centerless array fronts for the single and multi-frequency signals and compares them to the baseline Pareto-fronts. Particularly for the multi-frequency results, Figure 12 shows that there are several 9-element synthesized array configurations (including SA9-1 and SA9-3 shown in Figure 9) that outperformed the 36-element baseline Pareto-front. Note that the centerless 9-element synthesized array had a higher *H*-factor than the 36-element baseline for both the single and multi-frequency signals. Thus, the current study demonstrates how this approach to design arrays can enable increased array performance even when limited by the number of elements.

**Figure 13:**
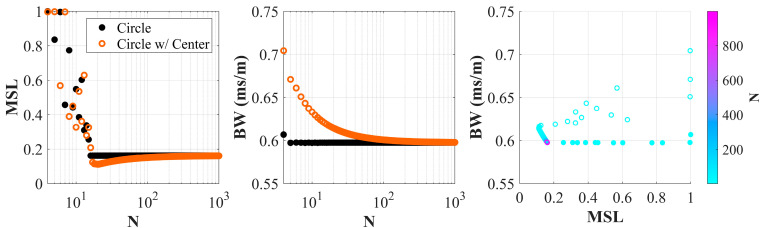
Performance of uniform circle array with and without a center element as a function of the total number of elements.

To further quantify the impact of a center element, the *BW* and *MSL* were computed for uniform circle arrays with and without center elements for varying total number of elements. The array aperture was fixed and the total number of elements ranged from 4 to 1000. For any given *N*, the arrays considered are an *N*-sided polygon without a center and an $\left(N-1\right)$-sided polygon with a center. Figure 13 shows the *MSL* and *BW* results from the same slowness region as above for a signal frequency of 20 Hz. The centerless array configuration reaches a consistent performance in *BW* at 5 elements and in *MSL* at 16 elements. For the center-element configurations, the *BW* performance never exceeds that of the centerless array, while the *MSL* is slightly better for *N* > 17. After about 100 elements, the performance of the two arrays has negligible variation.

**Figure 14:**
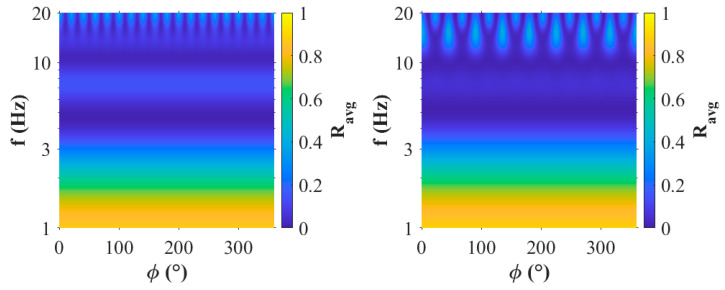
Average response of a 9-element uniform circle array (**left**) without and (**right**) with a center element within the slowness annulus of 320 to 420 m/s spanning signals from 1 to 20 Hz.

Furthermore, averaged response for a 9-element uniform circle array with and without a center element is provided in Figure 14. Here, the slowness grid of interest was narrowed to that considered in Wilson et al. [10] (i.e., 320 to 420 m/s). Here the average is computed within the annulus for each back azimuth, which shows the frequencies and back azimuths at which side lobes are likely in the annulus of interest. This was not used in the optimization process since it does not give a measure of *BW*. The frequency range considered spanned from 1 to 20 Hz (in 0.01 Hz increments from 1 to 3 Hz and 0.1 Hz increments from 3 Hz to 20 Hz). These results show that at frequencies above ~11 Hz side lobes are likely for the array with a center element at 45° increments starting at 0°. Conversely, the centerless array did not have side lobes of the same magnitude until signals above 15 Hz, which shows they occur in 20° increments.

## 5. Conclusions

Operationally effective passive sensing arrays must be designed considering many factors, including cost, space available, and frequencies of interest. For the current study, the array response pattern formed the basis for comparing different array designs. The array response was calculated considering a 2D array of omnidirectional elements measuring a monochromatic plane wave propagating in a locally homogeneous medium. The present work has treated array design as a multi-objective optimization problem balancing between maximizing the array spatial resolution (i.e., minimizing the main lobe beamwidth) and minimizing spatial aliasing (i.e., minimizing peak sidelobe level) and applying the concept of Pareto-efficiency to identify the optimal solutions. Using infrasound array design as an illustrated example, NSGA-II was implemented to generate species of array designs varying the number of elements (*N*), number of planes of symmetry (*S*), and whether there was a center element. The array designs were performed for either a single tone (8 Hz) or a multi-frequency signal (3, 8, 10, and 20 Hz) and compared relative to sample established array designs from the literature. After 1000 generations, the NSGA-II synthesized array designs outperformed the baseline arrays for most conditions. The one exception was the 9-element array with a single-tone, which highlighted that established array designs can be very effective at detection of signals within a narrow band and for such applications this approach is unlikely to be of much benefit.

For all cases considered, the centerless species outperformed the corresponding species (same signal and number of elements) with a center element. This indicates that in most scenarios, using a center element is not the most efficient use of that additional sensor. Furthermore, high-performing species with a lower number of elements had comparable performance to those of higher element designs (i.e., 9-element arrays had performance similar to the 36-element arrays). This was primarily quantified in terms of the *H*-factor that characterizes the Pareto-front. However, it should be noted that in practice, a researcher is not interested in the performance of the entire front. Rather, the researcher should select one design from the Pareto-front with that decision being based on the range of acceptable *BW* and *MSL* values for their application (e.g., an application might place a higher value on spatial resolution than spatial aliasing).

These results demonstrate a means of improving array performance for applications where increasing the number of sensors is difficult due to limited resources (e.g., cost, space, deployment time). This also highlights the fact that increasing the number of array elements has a diminishing return with respect to array performance. However, it should be noted that more elements always make the array more robust as the increased element number mitigates the impact of single-element failure. Future work should explore the effect of element number on the design problem, which could impact long-term planning for a given application.

## Figures and Tables

**Table 1:** Summary of the array design parameters (*N*, *S*, and center element), termed “species” from the genetic analogy, considered in the current demonstration.

*N*	*S*	Center	Elements/Segment
9	3	No	3
9	4	Yes	2
36	6	No	6
36	7	Yes	5
36	5	Yes	7

**Table 2:** List of baseline array configurations used as a reference when comparing the performance of the derived array configurations. The marker listed in the first column are the symbols used throughout the manuscript when plotting the performance of the given baseline configuration.

Marker	Configuration	9-Element	36-Element	Inputs
■	Uniform Grid	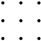	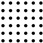	none
●	Uniformly spaced circle	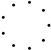	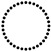	none
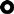	Uniformly spaced circle with center	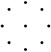	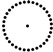	none
♦	Uniformly spaced concentric circle	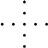	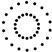	Inner circle diameter is half the outer diameter (aperture)
◀	Arcondoulis spiral	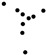	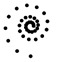	$r_{0}/r_{max}$ = 0.1, $\epsilon_{x}$$\;=\;1,\;\epsilon_{y}$ = 1, Φ = 11π/2
▲	Log-based multispiral	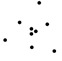	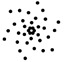	$r_{0}/r_{max}$ = 0.1 Φ = 5π/16 3 or 6 arms
▼	Underbrink multispiral	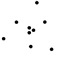	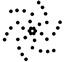	$r_{0}/r_{max}$ = 0.1 Φ = 5π/16 3 or 6 arms
★	VDCP (inner)	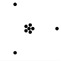	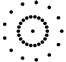	9-element: 5 inner, 3 outer 36-element: 22 inner, 13 outer
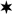	VDCP (outer)	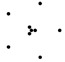	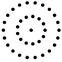	9-element: 3 inner, 5 outer 36-element: 13 inner, 22 outer

**Table 3:** Summary of the *H*-factor for the final Pareto-fronts for the synthesized and baseline arrays.

Signal	N	Species		H-Factor	
		Center	S	Synthesized	Baseline
8 Hz	36	no	6	73.6 ± 0.1%	69.6 ± 0.1%
		yes	7	72.6 ± 0.1%	
		yes	5	73.0 ± 0.1%	
	9	no	3	73.5 ± 0.1%	73.7 ± 0.1%
		yes	4	71.0 ± 0.1%	
Multi	36	no	6	69.4 ± 0.1%	65.5 ± 0.1%
		yes	7	68.6 ± 0.2%	
		yes	5	68.6 ± 0.1%	
	9	no	3	66.0 ± 0.2%	43.3 ± 0.2%
		yes	4	60.7 ± 0.2%	

## Data Availability

Data presented in this study is available upon reasonable request from the corresponding authors.
